# Genotypic diversity of multi- and pre-extremely drug-resistant *Mycobacterium tuberculosis* isolates from Morocco

**DOI:** 10.1371/journal.pone.0253826

**Published:** 2021-07-02

**Authors:** Amal Oudghiri, Ghizlane Momen, Achraf Aainouss, Amin Laglaoui, My Driss El Messaoudi, Mohammed El Mzibri, Imane Chaoui

**Affiliations:** 1 Department of Life Sciences, Medical and Biological Research Unit, National Center of Energy, Sciences and Nuclear Techniques, Rabat, Morocco; 2 Faculty of Sciences and Techniques, Biotechnology and Bimolecular Engineering Research Laboratory, Tangier, Morocco; 3 Laboratory of Mycabacteria, Pasteur Institute of Morocco, Casablanca, Morocco; 4 Faculty of Sciences, Laboratory of Microbiology Pharmacology, Biotechnology and Environment, Casablanca, Morocco; 5 Faculty of Sciences Ben M’Sik, Laboratory of Ecology and Environment, Casablanca, Morocco; St Petersburg Pasteur Institute, RUSSIAN FEDERATION

## Abstract

In Morocco, the prevalence of multidrug resistant tuberculosis (MDR-TB) continues to increase especially within previously treated cases; these MDR cases may evolve to extensively drug resistant tuberculosis (XDR-TB) raising major concern to TB control programs. From an epidemiological window, scarce informations are available about the genetic diversity of *Mycobacterium tuberculosis* (MTB) strains fueling these forms of resistance. The aim of this study was to assess to genetic diversity of MDR-MTB strains. Hence, this prospective study was conducted on patients diagnosed with MDR-TB at Pasteur Institute of Casablanca from 2010 to 2013. A total of 70 MDR-MTB isolates were genotyped by spoligotyping and 15-loci MIRU-VNTR methods. Spoligotyping generated four orphan patterns, five unique profiles whereas 61 strains were grouped in nine clusters (2 to 25 strains per cluster), the clustering rates being 87.1%. Subtyping by 15 loci MIRU-VNTR splitted all clusters already established by spoligotyping and generated 70 unique profiles not recognized in SITVIT2 database; clustering rate was equal to zero. HGDI analysis of 15 loci MIRU demonstrated that eight out of 15 loci were highly discriminant. Of note, all pre-XDR strains belongs to many clades, meaning that there no association between *gyrA* mutants and particular clade. Overall, the data generated by this study (i) describe the population structure of MDR MTBC in Morocco which is highly homogenous, (ii) confirm that TB in Morocco is almost exclusively transmitted by modern and evolutionary lineages with high level of biodiversity seen by MIRU, and (iii) validate the use of optimized 15-loci MIRU-VNTR format for future investigations in Morocco.

## Introduction

Worldwide, the emergence and transmission of drug resistance tuberculosis (DR-TB) especially multidrug resistant (MDR) and extremely drug resistant tuberculosis (XDR-TB) represents an enormous challenge to TB control programs [1]. In Morocco, new health crisis is emerging as TB epidemiology is radically changing due to increased population movements and migrations flow, subsequently leading to urgently design strategies to improve control and spread of DR-TB [2]. From an epidemiological window, a better understanding of Mycobacterium tuberculosis Complex (MTBC) population structure and the TB transmission dynamics within a community is crucial to avoid DR-TB spread at local and global scale [3].

Several studies reported correlation between MTB strain types and drug-resistant profiles and their contribution in understanding the origin and transmission patterns of drug-resistant strains; by combining two PCR-based methods, namely Spoligotyping (spacer oligonucleotide typing) which is based on the amplification and detection of the presence or absence of non-repetitive sequences called “spacers” between direct repeat elements of the MTBC genome [4,5]; and mycobacterial interspersed repetitive unit-variable number of tandem repeat (MIRU-VNTR) typing based on amplification of multiple loci (12, 15, or 24), using primers specific to each repeat locus, and on the determination of the sizes of the amplicons, which reflects the number of the targeted MIRU-VNTR copies [6,7].

Previous national studies based on spoligotyping and MIRU-VNTR typing (12 and 24 loci) were performed on random sets of MTB isolates to assess the genetic diversity of MTBC strains in different regions of Morocco [8–11]. Nevertheless, none of these investigations have characterized a significant proportion of MDR and pre-XDR MTB strains with accurate typing methods. Hence, the present study aims to (1) gain insight into genetic diversity of MDR MTB strains, (2) explore possible clonal expansion of particular lineages or sub-lineages and (3) find association between molecular fingerprint/clade and drug resistance profiles to assess to the population structure of drug resistant MTB strains.

## Material and methods

### Study population

A total of 70 MDR isolates were prospectively collected during four years’ period (2010 to 2013) in the laboratory of mycobacteria at Pasteur Institute, Casablanca.

#### Ethical approval

The study protocol was approved by the Ethics Committee of Institut Pasteur du Maroc (IPM2013-P3); the date of decision is Februry 18th, 2010. A written informed consent was also obtained from each study subject.

Each of the 70 isolates corresponds to a unique TB patient. Demographic and clinical information about patients including gender, age, and antecedent of TB were retrieved from laboratory records.

All the 70 M. *tuberculosis* isolates were already categorized as MDR-TB (defined as combined resistance to rifampin and isoniazid) and a subgroup was retained as pre-XDR (defined as MDR+ resistance to one of the fluoroquinolone + resistance to one of the three injectable drugs) based on drug susceptibility testing (DST) to first and second line drugs and sequencing of genes associated with resistant to rifampicin, isoniazid, fluoroquinolones, and injectable drugs [12].

### Sample preparation for strain typing

Crude DNA was prepared from scraped colonies in 400μl of distillated water and boiled at 100°C for 10–15 min to inactivate mycobacteria and release DNA. The crude DNA was stored at -20°C until use [13,14].

### Strain typing methods

The 70 isolates were subjected to conventional 43 spoligotyping and 15-loci MIRU-VNTR typing. Spoligotyping was performed, according to the manufacturer’s instructions, with the commercially available kit (Isogen Bioscience BV, Maarssen, The Netherlands), *M*. *tuberculosis* H37Rv and *M*. *bovis* BCG were used as control strains [5].

The 15-loci MIRU-VNTR typing for the 70 MDR-TB isolates was performed according to the protocol described by Le Flèche, using primers previously described by Supply *et al*, (2006) [6]. For each locus, DNA from *M*. *tuberculosis* H37Rv and deionized water were used as a positive and negative controls, respectively. PCR products were separated by electrophoresis on nusieve 3:1 agarose gel 1%, stained with ethidium bromide; the 50-bp molecular marker (leader IV, Life Biotechnologies) was used to determine the size of each PCR product.

### Data analysis

Spoligotypes in binary format were entered in an excel spreadsheet and compared to existing patterns in the international genotyping database SITVIT2 proprietary of Pasteur Institute of Guadeloupe and available online at http://www.pasteur-guadeloupe.fr:8081/SITVIT2/ [15]. Spoligotype patterns were assigned as SIT (spoligotype international type) if they share identical patterns already reported in SITVIT2 database. Clustered isolates were defined as having identical patterns generated by a typing method, “orphan” or “unknown” strains designate pattern not previously reported whereas “unique” spoligotypes corresponds to a SIT reported once in the study. Major MTB lineages and sublineages were assigned according to signatures provided in previous databases [16].

15-loci MIRU data were also entered in an excel sheet and compared to the international SITVIT2 database. MIT (MIRU international type) designates MIRU patterns shared by 2 or more isolates, whereas “not defined” represents patterns not already reported in the database.

The allelic diversity (h) of individual loci was calculated as th = 1 − ∑ xi 2 [n/(n − 1)], where xi is the frequency of the ith allele at the locus, and n the number of isolates [17,18].

The discriminatory power of a typing scheme was determined by the calculation of the Hunter and Gaston Discriminatory Index (HGDI) as reported earlier [19].

**Fig 1:**
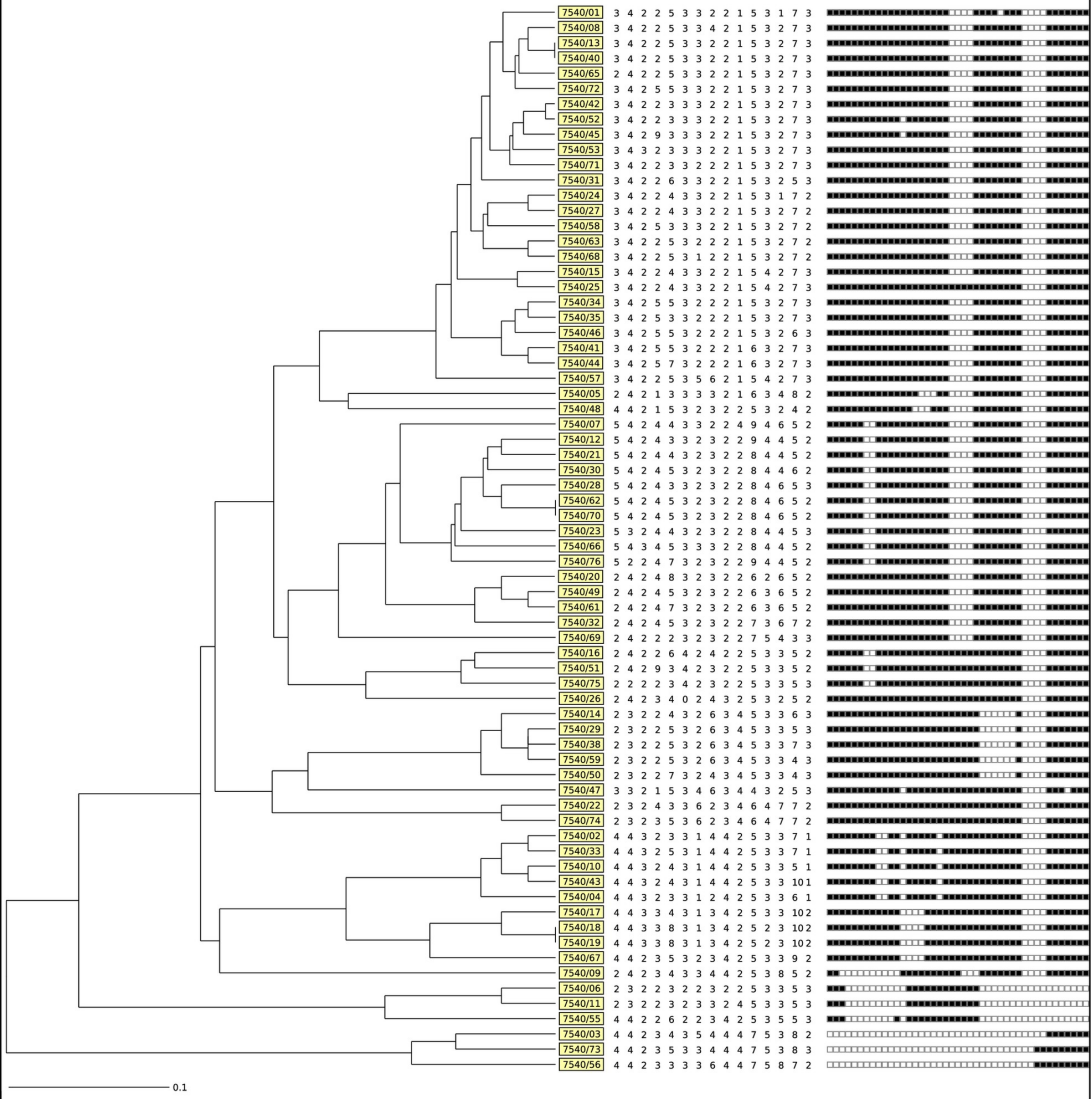
Phylogenetic tree based on spoligotyping and 15-locus MIRU-VNTR data of 70 MDR-MTB isolates from Grand Casablanca, Morocco. *A dendrogram was generated based on the UPGMA algorithm using tools available from the MIRU-VNTR*plus *identification database*. *From left to right*: *(1) UPGMA dendrogram generated by the 15-locus MIRU-VNTR*, *(2) spoligotyping patterns*, *and (3) strain number (boxed)*.

Molecular clustering of the isolates was determined by constructing a dendogram based on spoligotyping and MIRU-VNTR data (Fig 1).

## Results

### 1. Sociodemographic and clinical characteristics

A total of 70 MTB patients were included in this study with an age ranged from 18 to 50, the median age was 33.9 years. According to sex presentation, males were substantially more affected than females, with a sex ratio of 3.1. All MTB isolates were already phenotypically and genotypically characterized and retained as MDR-TB, each of the 70 isolates corresponded to a unique TB patient to allow accurate estimation of clustering rate.

The classifications based on history of previous TB treatment showed that only 2.9% of patients were new cases whereas 40% failed to treatment, 37.1% relapsed, 7.1% were loss to follow up, and clinical data were missing for 12.9% of the isolates.

All of the 70 isolates showed phenotypic resistance to both two major first line drugs: Rifampicin and Isoniazid. Along with DST results, genotyping data revealed the occurrence of mutations within *rpoB* and *katG* in all MTB isolates; Ser531Leu and Ser315Thr being the most predominant ones in *rpoB* and *katG* genes, respectively (S1 Table).

Second line drugs susceptibility testing showed that 15.7% were pre-XDR-TB isolates (11/70) of which 8.6% were resistant to Capreomycin (6/70), 1.4% resistant to Ofloxacin (1/70), 1.4% resistant to Amikacin (1/70) and 4.3% were resistant to Kanamycin (3/70); meanwhile no XDR-TB isolate was found. Along with DST for second line drugs, mutations in *gyrA* gene associated with resistance to FQs were reported in 25.7% of cases (18/70) and mutations in *eis* promoter region associated with resistance to injectable drugs were reported in 2.8% of strains (2/70).

### 2. Strain typing results

Spoligotyping of the 70 MDR-TB strains yielded 4 orphans patterns, 5 singletons while 61 strains were grouped in 9 clusters (2 to 25 strains per cluster) (Table 1). The clustering rate was 87.1%. The most prevalent spoligotypes in their respective families were SIT42/LAM9 (35.9%), SIT53/T1 (7.1%) and SIT47/H (7.1%). The repartition of MTB strains in the different clades was as follows: LAM (57.4%); T (18.5%); Haarlem (7.1%); S (7.1%); clade(s) not defined in SITVIT2 database (5.6%) and Beijing (4.3%). Of note, strains belonging to CAS and EAI clades were not found in this setting.

**Table 1:** Description of types generated by spoligotyping in this study by SIT and clade.

Spoligotyping pattern (binary format)	SIT	Lineage/sublineage	Total strains in this study (%)
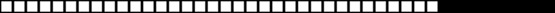	1	Beijing	2 (2.9)
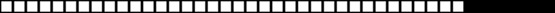	269	Beijing-like	1 (1.4)
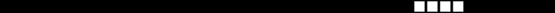	53	T1	5 (7.1)
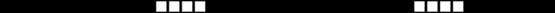	102	T	4 (5.7)
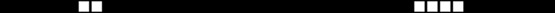	1069	T1	3 (4.3)
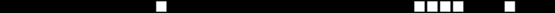	73	T2-T3	1 (1.4)
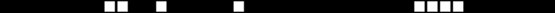	1068	S	5 (7.1)
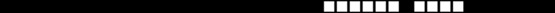	47	H1	5 (7.1)
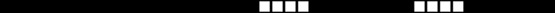	42	LAM9	25 (35.9)
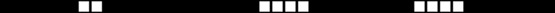	1064	LAM9	10 (14.4)
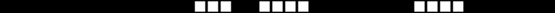	1075	LAM9	1 (1.4)
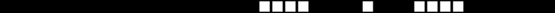	64	LAM6	1 (1.4)
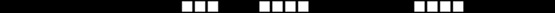	731	LAM9	1 (1.4)
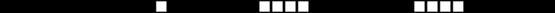	93	LAM5	2 (2.9)
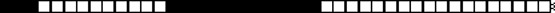	orphan	ND	1 (1.4)
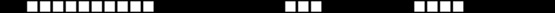	orphan	ND	1 (1.4)
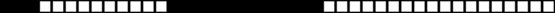	orphan	ND	1 (1.4)
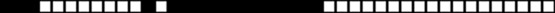	orphan	ND	1 (1.4)

ND: Not defined in SITVIT2 database.

Stratification of assigned spoligotyping families along with resistance profile was established (Table 2). With the exception of S clade which is made up of exclusively four MDR strains harboring mutations in *gyrA* gene, MDR as well pre-XDR strains belonged to many clades (Beijing, LAM, H and T). Of note, there was no significant association between the genotypes and age, gender, or specific drug susceptibility patterns.

**Table 2:** Distribution of clades according to drug resistance status.

Drug Resistance form	Spoligotype-assigned families of *M*. *tuberculosis* complex						Total
	Beijing	LAM	H	S	T	Undefined Clades/spoligotypes	
MDR	1	31	3	0	6	0	41
Pre-XDR	2	9	2	5	7	4	29

### MIRU-VNTR genotyping data

The MIRU set 15 loci was performed and generated 70 unique profile (S1 Table). All the pre-established clusters by spoligotyping were completely resolved by 15 loci MIRU-VNTR typing (Fig 1); clustering rate was equal to zero. An astonishing fact is that all patterns generated by MIRU were not previously reported in SITVIT2 database (S1 Table).

For allelic diversity of the MIRU loci, the discriminatory power was calculated using HGDI (Table 3). We note that Mtub04, MIRU40, MIRU10, Mtub21, QUB11b and Mtub30 loci were highly discriminative (HGDI > 0.60) whereas the remaining loci were less discriminative (HGDI < 0.60). The discriminatory power of 15-loci MIRU-VNTRs format being 1 vs. 0.84 for spoligotyping.

**Table 3:** Allelic polymorphism of 15 MIRU loci assessed on 70 MDR MTB strains.

MIRU Locus	Allele number											Allelic diversity*
	0	1	2	3	4	5	6	7	8	9	10	
Mtub04			22	25	13	10						0,7292
ETRC			2	11	57							0,3159
MIRU04			61	9								0,2273
MIRU40		3	31	10	16	7				2		0,7208
MIRU10			1	19	13	27	3	4	3			0,7466
MIRU16	1		3	63	3							0,1888
Mtub21		8	34	23	1	2	2					0,6505
QUB11b			27	26	10		7					0,6928
ETRA			48	9	13							0,4857
Mtub30		26	31		13							0,6406
MIRU26					1	45	8	6	7	3		0,5623
MIRU31			3	48	15	4						0,4857
Mtub39		2	26	20	8	1	9	2	2			0,759
QUB26				1	3	25	4	30	3	1	3	0,6894
QUB4156		4	32	34								0,5598

*The allelic diversity of the loci was classified as highly (HGDI > 0.6), moderately (0.3< HGI < 0.6) and poorly discriminative (HGI < 0.3), according to Sola et al. (2003) [20].

## Discussion

To our knowledge, this is the first molecular characterization study to gain insight on MDR and pre-XDR MTB population structure in Casablanca, recognized as a hot spot area of TB in Morocco. Indeed, few previous national studies carried out from 1997 to 2013 have addressed the question of MTBC genetic diversity in Morocco but were performed either on small sample size, or on biased samples in terms of susceptibility/drug resistant status, or by genotyping method(s) with moderately discriminatory power which often overestimates the clustering and recent transmission rates [8–11].

Clinical data showed that this sample collection is dominated by high relapse and failure rates as only 2.9% of the 70 MDR TB were newly diagnosed cases. Up to 90.4% (66/70) MDR strains harbored the most common mutations in *rpoB* (Ser531Leu, His526Tyr and Asp516Tyr) and *katG* (Ser315Thr) genes associated with RIF and INH resistance respectively.

Genotypic analysis by spoligotyping revealed a population structure dominated by Euro-American lineages, namely LAM, Haarlem and T belonging to the ‘‘evolutionary recent”TbD1-/PGG2/3 phylogenetic group. The MTB population structure is highly homogeneous and dominated by LAM lineage with three (LAM5, LAM6 and LAM9) out of its twelve reported sublineages throughout the world, LAM9 with its prototype SIT42 being the most prevalent. These findings corroborate previous reports confirming that LAM is a hallmark of MTBC population structure in Morocco [3].

Regarding the association of MTBC lineages and MDR/pre-XDR genotypes, a previous preliminary study reported the predominance of a single ubiquitous clade (LAM) within the pre-XDR MTB, resistant to FQs and harboring mutations in *gyrA* gene [21] in contrast to the results from this study demonstrating that there was no association between clades and DR genotypes as almost strains from different lineages/sublineages were involved in both MDR and pre-XDR TB except S clade which is more than being rare, was made up exclusively with MDR strains with unique SIT (1068), this clade deserves more investigations to confirm or infirm its endemicity and its strong association with MDR genotype [21,22].

In fact, it has been reported that the association of certain strain families with pre-XDR/XDR-TB is probably due to their enhanced intrinsic capacity to acquire DR to FQs and/or injectable drugs, or to their relatively more effective transmission as MDR-TB [23].

Although Spoligotyping has enhanced our understanding of global strain distribution and has allowed assignment of SITs to track MTB strains worldwide, it is unlikely an unreliable tool to study TB transmission dynamics as it overestimates, very often, links between MTB patients; clustering rates being 87.1% from this study. Also, Spoligotyping is prone to homoplasy (identical spoligotypes in phylogenetically unrelated strains) [24].

Numerous studies reported that MIRU-VNTR is more suitable for epidemiological surveillance as it allows a deeper discrimination between MTB isolates. Our results revealed a high level a biodiversity within MTB isolates seen by MIRU-VNTR typing. In contrast to spoligotyping which clustered the majority of the strains, MIRU split all the clusters; also an astonishing fact is that all patterns generated by MIRU were not previously reported as a MIT. Also, the discriminatory power of spoligotyping used alone (HGDI 0.87) significantly increased when spoligotyping was combined with 15-loci MIRUs (HGDI 1), thus our data argue the use of 15-loci MIRUs for epidemiological studies, this scheme may be improved by excluding one or more MIRU loci with a poor discriminatory power and including MIRU23 for example, the latter has been shown to exhibit high allelic diversity among MTB isolates from Morocco [8–11]. Hopefully, future analyses with an optimized set of MIRU will probably add value to current typing scheme in our setting for epidemiological purposes whereas 24-locus MIRU-VNTR would serve for phylogenetic studies.

Numerous reports have documented the association of Beijing strains with MDR-TB profile [25,26]. In Morocco, and despite their presence, they are not exclusively associated with MDR-TB; their low prevalence in Morocco remains too speculative.

In addition, no association between particular clade/MDR Pre-XDR genotype was detected. In contrast to data reported by Chaoui et al. (2018) suggestive of possible emergence of genotype/lineage clone; our data failed to find any association between specific clade and genetic mutations conferring resistance to FQs or injectable drugs [3].

The present study constitutes the first attempt to address MDR MTB diversity in Morocco. Based on these preliminary findings, conclusions made about the transmission dynamics of TB within MDR MTB isolates would be wrong because of the heterogeneity of isolates in terms of panel size, drug resistance status, origin of isolates (only one hot spot area); new vs pretreated cases and lack of information about epidemiologic links between patients. Therefore, this study is not conclusive about the association between genotypes/lineages observed Vs. occurrence of drug-resistance and type of disease (new cases vs relapse, failure, chronic tuberculosis, etc…) and highlights the need for further studies on large number of resistant isolates (MDR/Pre-XDR and XDR), representative of all hot spot areas of TB in Morocco, to provide a more accurate picture of the epidemiology of drug-resistant MTB strains in Morocco and to determine the rate of recent transmission among the population.

These findings highlight the relevance of proper infection control, as well as effective treatment, to further contain highly developed DR-TB. Moreover, further studies are urgently needed to prospectively identify the transmission route through contact tracing and real-time DNA fingerprinting, to estimate the evolution of MTB population over the time; to detect important demographic events in MTBC history such as, for instance, episodes of expansion of the Beijing lineage widely associated to massive spread of MDR-TB strains [27].

Beyond classical spoligotyping and MIRU-VNTR typing techniques, the application of modern genotyping methods such as whole Genome Sequencing has shown a big potential in the analysis and identification of outbreak-related transmission chains, and ultimately would be the accurate tool to design and compare research studies for public health worldwide [28–30]. For instance, molecular typing is performed mostly in developed countries, rarely in areas of TB endemicity where routine molecular epidemiological surveillance is crucial to reduce TB transmission within a community.

## Conclusion

In summary, the present study offers the first insight about the genetic diversity of MDR- and Pre-XDR MTB strains from a high incidence area of TB in Morocco, the study confirms that TB is almost exclusively transmitted by modern and evolutionary lineages with extreme level of biodiversity seen by MIRU. Of course, additional studies on larger panel of isolates from the whole country would be of great value to establish the genetic landscape and to interrupt chain of transmission. Hopefully, an accurate and a simpler model to survey tuberculosis transmission in Morocco particularly MDR and XDR TB would be a great value.

## Supporting information

S1 Fig Phylogenetic tree excluding spoligo profiles. (PDF)

S2 Fig Phylogenetic tree including reference strains. (PDF)

S1 Table Detailed results regarding demographic, drug resistance & associated mutations and strain typing on 70 *M*. *tuberculosis* strains isolated in Grand Casablanca, Morocco. (DOCX)

S1 File Combined MIRU VNTR results (scheme 15). (XLSX)

## References

[1] World Health Organization. Global tuberculosis report. 2018. WHO (2018) “Global Tuberculosis control: WHO report 2018.” Geneva, Switzerland: World Health Organization: 231. Available: https://apps.who.int/iris/handle/10665/274453. Accessed 2020 June 19.

[2] P.N.L.A.T.(2018). Plan national de lutte antituberculeuse au Maroc. Brochure progrès défis de la Tuberculose.

[3] Chaoui I, Oudghiri A, EL Mzibri M. Molecular Epidemiology of Tuberculosis and Transmission Dynamics of Mycobacterium tuberculosis Complex in Morocco. J Mycobact Dis. 2018; 8: 268. doi: 10.4172/2161-1068.1000268

[4] Groenen PM, Bunschoten AE, van Soolingen D, van Embden JD. Nature of DNA polymorphism in the direct repeat cluster of Mycobacterium tuberculosis; application for strain differentiation by a novel typing method. Mol Microbiol. 1993;10(5):1057–1065. doi: 10.1111/j.1365-2958.1993.tb00976.x 7934856

[5] Kamerbeek J, Schouls L, Kolk A., Van Agterveld M, van Soolingen D, Kuijper S, et al. Simultaneous detection and strain differentiation of Mycobacterium tuberculosis for diagnosis and epidemiology. J Clin Microbiol. 1997;35(4):907–914. doi: 10.1128/jcm.35.4.907-914.1997 9157152 PMC229700

[6] Supply P, Allix C, Lesjean S, Cardoso-Oelemann M, Rüsch-Gerdes S, Willery E, et al. Proposal for standardization of optimized mycobacterial interspersed repetitive unit-variable-number tandem repeat typing of Mycobacterium tuberculosis. J Clin Microbiol. 2006;44(12):4498–4510. doi: 10.1128/JCM.01392-06 17005759 PMC1698431

[7] Supply P, Lesjean S, Savine E, Kremer K, van Soolingen D, Locht C. Automated high-throughput genotyping for study of global epidemiology of Mycobacterium tuberculosis based on mycobacterial interspersed repetitive units. J Clin Microbiol. 2001;39(10):3563–3571. doi: 10.1128/JCM.39.10.3563-3571.2001 11574573 PMC88389

[8] Tazi L, Reintjes R, Bañuls AL. Tuberculosis transmission in a high incidence area: A retrospective molecular epidemiological study of Mycobacterium tuberculosis in Casablanca, Morocco. Infect Genet Evol. 2007;7(5):636–644. doi: 10.1016/j.meegid.2007.06.005 17689298

[9] Lahlou O, Millet J, Chaoui I, Sabouni R, Filali-Maltouf A, Akrim M, et al. The genotypic population structure of Mycobacterium tuberculosis complex from Moroccan patients reveals a predominance of Euro-American lineages. PLoS One. 2012;7(10):e47113. doi: 10.1371/journal.pone.0047113 23077552 PMC3471964

[10] Chaoui I, Zozio T, Lahlou O, Sabouni R, Abid M, El Aouad R. Contribution of spoligotyping and MIRU-VNTRs to characterize prevalent Mycobacterium tuberculosis genotypes infecting tuberculosis patients in Morocco. Infect Genet Evol. 2014;21:463–471. doi: 10.1016/j.meegid.2013.05.023 23732366

[11] Bouklata N, Supply P, Jaouhari S, Charof R, Seghrouchni F, Sadki K, et al. Molecular typing of Mycobacterium tuberculosis complex by 24-locus based MIRU-VNTR typing in conjunction with spoligotyping to assess genetic diversity of strains circulating in Morocco. PLoS One. 2015;10(8):e0135695. Published 2015 Aug 18. doi: 10.1371/journal.pone.0135695 26285026 PMC4540494

[12] Oudghiri A, Karimi H, Chetioui F, Zakham F, Bourkadi JE, El Messaoudi MD, et al. Molecular characterization of mutations associated with resistance to second-line tuberculosis drug among multidrug-resistant tuberculosis patients from high prevalence tuberculosis city in Morocco. BMC Infect Dis. 2018;18(1):98. Published 2018 Feb 27. doi: 10.1186/s12879-018-3009-9 29486710 PMC5830342

[13] Victor TC, Warren R, Butt JL, Jordaan AM, Felix JV, Venter A, et al. Genome and MIC stability in Mycobacterium tuberculosis and indications for continuation of use of isoniazid in multidrug-resistant tuberculosis. J Med Microbiol. 1997;46(10), 847–857. doi: 10.1099/00222615-46-10-847 9364141

[14] Chaoui I, Sabouni R, Kourout M, Jordaan AM, Lahlou O, Elouad R, et al. Analysis of isoniazid, streptomycin and ethambutol resistance in *Mycobacterium tuberculosis* isolates from Morocco. J Infect Dev Ctries. 2009;3(4):278–284. Published 2009 May 1. doi: 10.3855/jidc.125 19759491

[15] Demay C, Liens B, Burguière T, Hill V, Couvin D, Millet J, et al. SITVITWEB—a publicly available international multimarker database for studying *Mycobacterium tuberculosis* genetic diversity and molecular epidemiology. Infect Genet Evol. 2012;12(4):755–766. doi: 10.1016/j.meegid.2012.02.004 22365971

[16] Brudey K, Driscoll JR, Rigouts L, Prodinger WM, Gori A, Al-Hajoj SA, et al. Mycobacterium tuberculosis complex genetic diversity: mining the fourth international spoligotyping database (SpolDB4) for classification, population genetics and epidemiology. BMC Microbiol. 2006;6:23. Published 2006 Mar 6. doi: 10.1186/1471-2180-6-23 16519816 PMC1468417

[17] Selander RK, Caugant DA, Ochman H, Musser JM, Gilmour MN, Whittam TS. Methods of multilocus enzyme electrophoresis for bacterial population genetics and systematics. Appl Environ Microbiol. 1986;51(5):873–884. doi: 10.1128/aem.51.5.873-884.1986 2425735 PMC238981

[18] Small, P. M., and J. D. A. van Embden.1994. Molecular epidemiology of tuberculosis, p. 569–582.InB. R. Bloom (ed.), Tuberculosis: pathogenesis, protection and control. American Society for Microbiology, Washington, D.C.

[19] Hunter PR, Gaston MA. Numerical index of the discriminatory ability of typing systems: an application of Simpson’s index of diversity. J Clin Microbiol. 1988;26(11):2465–2466. doi: 10.1128/jcm.26.11.2465-2466.1988 3069867 PMC266921

[20] Sola C, Filliol I, Legrand F, Lesjean S, Locht C, Supply P, et al. Genotyping of the Mycobacterium tuberculosis complex using MIRUs: association with VNTR and spoligotyping for molecular epidemiology and evolutionary genetics. Infect Genet Evol. 2003;3(2):125–133. doi: 10.1016/s1567-1348(03)00011-x 12809807

[21] Chaoui I, Oudghiri A, EL Mzibri M. Characterization of *gyrA* and *gyrB* mutations associated with fluoroquinolones resistance in *Mycobacterium tuberculosis* isolates from Morocco. J Glob Antimicrob Resist. 2018;12:171–174. doi: 10.1016/j.jgar.2017.10.003 29033301

[22] Valcheva V, Mokrousov I, Panaiotov S, et al. Bulgarian specificity and controversial phylogeography of *Mycobacterium tuberculosis* spoligotype ST 125__BGR. FEMS Immunol Med Microbiol. 2010;59(1):90–99. doi: 10.1111/j.1574-695X.2010.00667.x 20402768

[23] Chihota VN, Müller B, Mlambo CK, Pillay M, Tait M, Streicher EM, et al. Population structure of multi-and extensively drug-resistant *Mycobacterium tuberculosis* strains in South Africa. J Clin Microbiol. 2012;50(3):995–1002. doi: 10.1128/JCM.05832-11 22170931 PMC3295122

[24] Tulu B, Ameni G. Spoligotyping based genetic diversity of Mycobacterium tuberculosis in Ethiopia: a systematic review. BMC Infect Dis. 2018;18(1):140. Published 2018 Mar 27. doi: 10.1186/s12879-018-3046-4 29587640 PMC5870191

[25] Wang XH, MA AG, Han XX, Gu XM, Fu LP, Li PG. Correlations between drug resistance of Beijing/W lineage clinical isolates of Mycobacterium tuberculosis and sublineages: A 2009–2013 prospective study in Xinjiang province, China. Med Sci Monit. 2015;21:1313–1318. Published 2015 May 7. doi: 10.12659/MSM.892951 25950148 PMC4434980

[26] Liu Q, Luo T, Dong X, Sun G, Liu Z, Gan M, et al. Genetic features of Mycobacterium tuberculosis modern Beijing sublineage. Emerg Microbes Infect. 2016;5(2):e14. Published 2016 Feb 24. doi: 10.1038/emi.2016.14 26905026 PMC4777927

[27] Merker M, Blin C, Mona S, Duforet-Frebourg N, Lecher S, Willery E, et al. Evolutionary history and global spread of the Mycobacterium tuberculosis Beijing lineage. Nat Genet. 2015;47(3):242–249. doi: 10.1038/ng.3195 25599400 PMC11044984

[28] Schürch AC, van Soolingen D. DNA fingerprinting of Mycobacterium tuberculosis: from phage typing to whole-genome sequencing. Infect Genet Evol. 2012;12(4):602–609. doi: 10.1016/j.meegid.2011.08.032 22067515

[29] Walker TM, Ip CL, Harrell RH, Evans JT, Kapatai G, Dedicoat MJ. Whole-genome sequencing to delineate Mycobacterium tuberculosis outbreaks: a retrospective observational study. Lancet Infect Dis. 2013;13(2):137–146. doi: 10.1016/S1473-3099(12)70277-3 23158499 PMC3556524

[30] Abascal E, Pérez-Lago L, Martínez-Lirola M, Chiner-Oms Á, Herranz M, Chaoui I, et al. Whole genome sequencing–based analysis of tuberculosis (TB) in migrants: rapid tools for cross-border surveillance and to distinguish between recent transmission in the host country and new importations. Euro Surveill. 2019;24(4):1800005. doi: 10.2807/1560-7917.ES.2019.24.4.1800005 30696526 PMC6351995

