# Supplementary figures and images for: Genotypic diversity of multi- and pre-extremely drug-resistant Mycobacterium tuberculosis isolates from Morocco
Source: PLoS One. 2021 Jul 2;16(7):e0253826. doi: 10.1371/journal.pone.0253826 (PMC8253442; doi:10.1371/journal.pone.0253826)

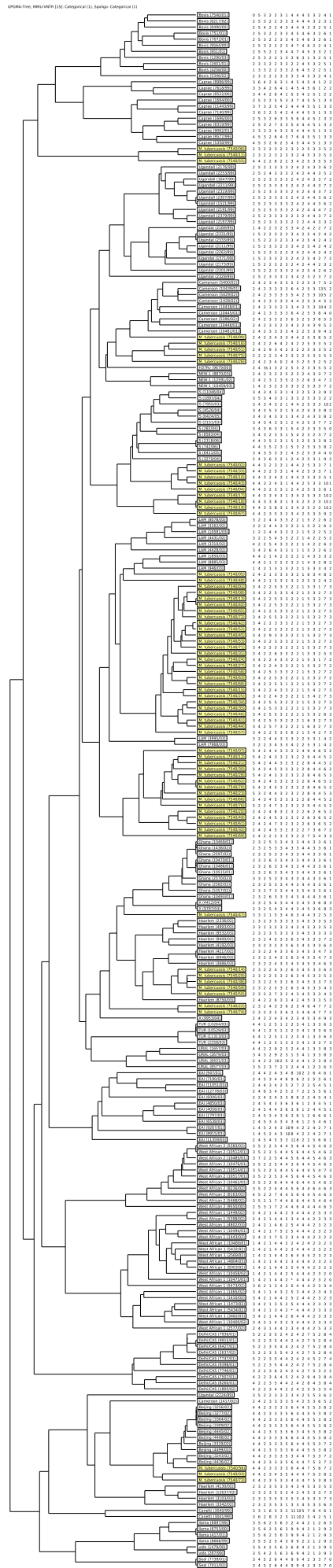

Supplement: S1 Fig — (PDF) [file pone.0253826.s001.pdf]

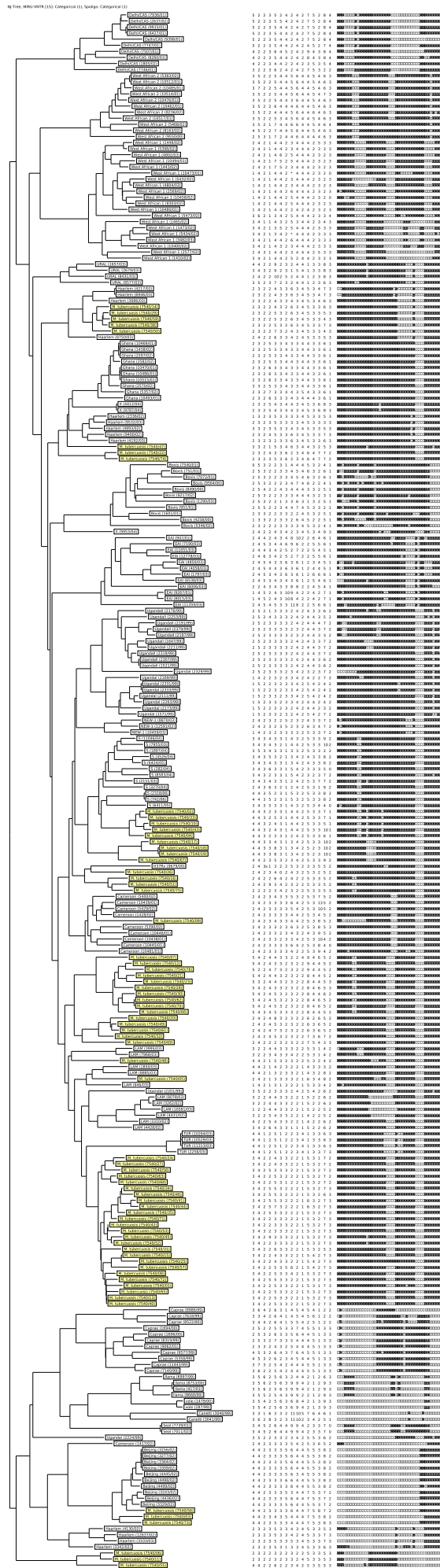

Supplement: S2 Fig — (PDF) [file pone.0253826.s002.pdf]
