# Supplementary material for: Genotypic diversity of multi- and pre-extremely drug-resistant Mycobacterium tuberculosis isolates from Morocco
Source: PLoS One. 2021 Jul 2;16(7):e0253826. doi: 10.1371/journal.pone.0253826 (PMC8253442; doi:10.1371/journal.pone.0253826)
Supplement: S1 Table — (DOCX) [file pone.0253826.s003.docx]

S1 Table: Detailed results regarding demographic, drug resistance & associated mutations and strain typing on 70 M. tuberculosis strains isolated in Grand Casablanca, Morocco

| ID | Sexe/  Age | City | Clinical data | RIF | INH | KAN | OFX | AMK | CAP | *rpoB* | KatG | *gyrA* | *gyrB* | *rrs* | *tlyA* | *eis* | DST | Spoligo pattern (Binary description) |
| --- | --- | --- | --- | --- | --- | --- | --- | --- | --- | --- | --- | --- | --- | --- | --- | --- | --- | --- |
| 1 | M/NA | casa | Treatment after failure | R | R |  |  |  |  | TCG531TTG | AGC315ACC | *wt* | *wt* | *wt* | A33/G | *wt* | 2 | ⬛⬛⬛⬛⬛⬛⬛⬛⬛⬛⬛⬛⬛⬛⬛⬛⬛⬛⬛⬛⬜⬜⬜⬜⬛⬛⬛⬛⬜⬛⬛⬛⬜⬜⬜⬜⬛⬛⬛⬛⬛⬛⬛ |
| 2 | F/36 | casa | Relapse | R | R |  |  |  |  | CAC526TGC | AGC315ACC | GCG90GTG | *wt* | *wt* | A33/G | *wt* | 3 | ⬛⬛⬛⬛⬛⬛⬛⬛⬜⬜⬛⬛⬜⬛⬛⬛⬛⬛⬜⬛⬛⬛⬛⬛⬛⬛⬛⬛⬛⬛⬛⬛⬜⬜⬜⬜⬛⬛⬛⬛⬛⬛⬛ |
| 3 | F/38 | casa | Treatment after failure | R | R | S | S | S | S | GAC516GTC | AGC315ACC | GAC94GCC; ACC95AGC | *wt* | *wt* | A33/G | *wt* | 3 | ⬜⬜⬜⬜⬜⬜⬜⬜⬜⬜⬜⬜⬜⬜⬜⬜⬜⬜⬜⬜⬜⬜⬜⬜⬜⬜⬜⬜⬜⬜⬜⬜⬜⬜⬜⬜⬛⬛⬛⬛⬛⬛⬛ |
| 4 | M/NA | casa | Relapse | R | R | S | S | S | R | TCG531TTG | AGC315ACC | TCG91CCG | *wt* | *wt* | A33/G | *wt* | 3 | ⬛⬛⬛⬛⬛⬛⬛⬛⬜⬜⬛⬛⬜⬛⬛⬛⬛⬛⬜⬛⬛⬛⬛⬛⬛⬛⬛⬛⬛⬛⬛⬛⬜⬜⬜⬜⬛⬛⬛⬛⬛⬛⬛ |
| 5 | F/34 | casa | Relapse | R | R | S | S | S | R | TCG531TTG | AGC315ACC | ACC95AGC | *wt* | *wt* | A33/G | *wt* | 3 | ⬛⬛⬛⬛⬛⬛⬛⬛⬛⬛⬛⬛⬛⬛⬛⬜⬜⬜⬛⬛⬜⬜⬜⬜⬛⬛⬛⬛⬛⬛⬛⬛⬜⬜⬜⬜⬛⬛⬛⬛⬛⬛⬛ |
| 6 | F /35 | casa | Treatment after failure | R | R |  |  |  |  | TCG531TTG | AGC315ACC | GAC94GCC; ACC95AGC | *wt* | *wt* | A33/G | *wt* | 3 | ⬛⬛⬛⬜⬜⬜⬜⬜⬜⬜⬜⬜⬜⬛⬛⬛⬛⬛⬛⬛⬛⬛⬛⬛⬛⬜⬜⬜⬜⬜⬜⬜⬜⬜⬜⬜⬜⬜⬜⬜⬜⬜⬜ |
| 7 | M/33 | casa | Treatment after failure | R | R | S | S | S | S | TCG531TTG | AGC315ACC | ACC95AGC | *wt* | *wt* | A33/G | *wt* | 2 | ⬛⬛⬛⬛⬛⬛⬜⬜⬛⬛⬛⬛⬛⬛⬛⬛⬛⬛⬛⬛⬜⬜⬜⬜⬛⬛⬛⬛⬛⬛⬛⬛⬜⬜⬜⬜⬛⬛⬛⬛⬛⬛⬛ |
| 8 | M/18 | casa | Treatment after failure | R | R | S | S | S | S | TCG531TTG | AGC315ACC | *wt* | *wt* | *wt* | A33/G | *wt* | 2 | ⬛⬛⬛⬛⬛⬛⬛⬛⬛⬛⬛⬛⬛⬛⬛⬛⬛⬛⬛⬛⬜⬜⬜⬜⬛⬛⬛⬛⬛⬛⬛⬛⬜⬜⬜⬜⬛⬛⬛⬛⬛⬛⬛ |
| 9 | M/50 | casa | Treatment after failure | R | R |  |  |  |  | GAC516GTC | AGC315ACC | GAC94GCC | *wt* | *wt* | A33/G | *wt* | 3 | ⬛⬛⬜⬜⬜⬜⬜⬜⬜⬜⬜⬜⬛⬛⬛⬛⬛⬛⬛⬛⬛⬛⬜⬜⬜⬛⬛⬛⬛⬛⬛⬛⬜⬜⬜⬜⬛⬛⬛⬛⬛⬛⬛ |
| 10 | M/28 | casa | loss to follow-up | R | R | S | S | S | R | TCG531TTG | AGC315ACC | TCG91CCG | *wt* | *wt* | A33/G | *wt* | 3 | ⬛⬛⬛⬛⬛⬛⬛⬛⬜⬜⬛⬛⬜⬛⬛⬛⬛⬛⬜⬛⬛⬛⬛⬛⬛⬛⬛⬛⬛⬛⬛⬛⬜⬜⬜⬜⬛⬛⬛⬛⬛⬛⬛ |
| 11 | F/NA | casa | loss to follow-up | R | R | S | S | S | S | TCG531TTG | AGC315ACC | GAC94GCC | *wt* | *wt* | A33/G | *wt* | 3 | ⬛⬛⬛⬜⬜⬜⬜⬜⬜⬜⬜⬜⬜⬛⬛⬛⬛⬛⬛⬛⬛⬛⬛⬛⬛⬜⬜⬜⬜⬜⬜⬜⬜⬜⬜⬜⬜⬜⬜⬜⬜⬜⬜ |
| 12 | M/NA | casa | Relapse | R | R | S | S | S | S | TCG531TTG | AGC315ACC | ACC95AGC | *wt* | *wt* | A33/G | *wt* | 2 | ⬛⬛⬛⬛⬛⬛⬜⬜⬛⬛⬛⬛⬛⬛⬛⬛⬛⬛⬛⬛⬜⬜⬜⬜⬛⬛⬛⬛⬛⬛⬛⬛⬜⬜⬜⬜⬛⬛⬛⬛⬛⬛⬛ |
| 13 | M/NA | casa | loss to follow-up | R | R | S | S | S | S | TCG531TTG | AGC315ACC | ACC95AGC | *wt* | *wt* | A33/G | *wt* | 2 | ⬛⬛⬛⬛⬛⬛⬛⬛⬛⬛⬛⬛⬛⬛⬛⬛⬛⬛⬛⬛⬜⬜⬜⬜⬛⬛⬛⬛⬛⬛⬛⬛⬜⬜⬜⬜⬛⬛⬛⬛⬛⬛⬛ |
| 14 | M/33 | casa | Relapse | R | R | S | S |  | S | TCG531TTG | AGC315ACC | TCG91CCG | *wt* | *wt* | A33/G | *wt* | 3 | ⬛⬛⬛⬛⬛⬛⬛⬛⬛⬛⬛⬛⬛⬛⬛⬛⬛⬛⬛⬛⬛⬛⬛⬛⬛⬜⬜⬜⬜⬜⬜⬛⬜⬜⬜⬜⬛⬛⬛⬛⬛⬛⬛ |
| 15 | M/46 | casa | Relapse | R | R | S |  | S |  | TCG531TTG | AGC315ACC | ACC95AGC | *wt* | *wt* | A33/G | *wt* | 2 | ⬛⬛⬛⬛⬛⬛⬛⬛⬛⬛⬛⬛⬛⬛⬛⬛⬛⬛⬛⬛⬜⬜⬜⬜⬛⬛⬛⬛⬛⬛⬛⬛⬜⬜⬜⬜⬛⬛⬛⬛⬛⬛⬛ |
| 16 | M/NA | casa | Treatment after failure | R | R | S | S | S | S | TCG531TTG | AGC315ACC | *wt* | *wt* | *wt* | A33/G | *wt* | 2 | ⬛⬛⬛⬛⬛⬛⬜⬜⬛⬛⬛⬛⬛⬛⬛⬛⬛⬛⬛⬛⬛⬛⬛⬛⬛⬛⬛⬛⬛⬛⬛⬛⬜⬜⬜⬜⬛⬛⬛⬛⬛⬛⬛ |
| 17 | M/NA | casa | Relapse | R | R | S | S | S | R | TCG531TTG | AGC315ACC | ACC95AGC | *wt* | *wt* | A33/G | *wt* | 3 | ⬛⬛⬛⬛⬛⬛⬛⬛⬛⬛⬛⬛⬜⬜⬜⬜⬛⬛⬛⬛⬛⬛⬛⬛⬛⬛⬛⬛⬛⬛⬛⬛⬜⬜⬜⬜⬛⬛⬛⬛⬛⬛⬛ |
| 18 | F/22 | casa | Treatment after failure | R | R | S | S | S | S | TCG531TTG | AGC315ACC | GAC94GCC | *wt* | *wt* | A33/G | *wt* | 3 | ⬛⬛⬛⬛⬛⬛⬛⬛⬛⬛⬛⬛⬜⬜⬜⬜⬛⬛⬛⬛⬛⬛⬛⬛⬛⬛⬛⬛⬛⬛⬛⬛⬜⬜⬜⬜⬛⬛⬛⬛⬛⬛⬛ |
| 19 | F/22 | casa | Treatment after failure | R | R | S | S | S | S | TCG531TTG | AGC315ACC | GAC94GCC | *wt* | *wt* | A33/G | *wt* | 3 | ⬛⬛⬛⬛⬛⬛⬛⬛⬛⬛⬛⬛⬜⬜⬜⬜⬛⬛⬛⬛⬛⬛⬛⬛⬛⬛⬛⬛⬛⬛⬛⬛⬜⬜⬜⬜⬛⬛⬛⬛⬛⬛⬛ |
| 20 | M/32 | casa | Relapse | R | R | S | S | S | S | TCG531TTG | AGC315ACC | *wt* | *wt* | *wt* | A33/G | *wt* | 2 | ⬛⬛⬛⬛⬛⬛⬛⬛⬛⬛⬛⬛⬛⬛⬛⬛⬛⬛⬛⬛⬜⬜⬜⬜⬛⬛⬛⬛⬛⬛⬛⬛⬜⬜⬜⬜⬛⬛⬛⬛⬛⬛⬛ |
| 21 | M/46 | casa | Relapse | R | R | S | S | S | S | TCG531TTG | AGC315ACC | *wt* | *wt* | *wt* | A33/G | *wt* | 2 | ⬛⬛⬛⬛⬛⬛⬜⬜⬛⬛⬛⬛⬛⬛⬛⬛⬛⬛⬛⬛⬜⬜⬜⬜⬛⬛⬛⬛⬛⬛⬛⬛⬜⬜⬜⬜⬛⬛⬛⬛⬛⬛⬛ |
| 22 | M/25 | casa | Treatment after failure | R | R | S | S | S | S | GAC516TAC | AGC315ACC | *wt* | *wt* | *wt* | A33/G | *wt* | 2 | ⬛⬛⬛⬛⬛⬛⬛⬛⬛⬛⬛⬛⬛⬛⬛⬛⬛⬛⬛⬛⬛⬛⬛⬛⬛⬛⬛⬛⬛⬛⬛⬛⬜⬜⬜⬜⬛⬛⬛⬛⬛⬛⬛ |
| 23 | M/NA | casa | loss to follow-up | R | S | S | S | S | S | TCG531TTG | AGC315ACC | GCG90GTG | *wt* | *wt* | A33/G | *wt* | 3 | ⬛⬛⬛⬛⬛⬛⬜⬜⬛⬛⬛⬛⬛⬛⬛⬛⬛⬛⬛⬛⬜⬜⬜⬜⬛⬛⬛⬛⬛⬛⬛⬛⬜⬜⬜⬜⬛⬛⬛⬛⬛⬛⬛ |
| 24 | M/50 | casa | Relapse | R | R | S | S | S | S | TCG531TTG | AGC315ACC | *wt* | *wt* | *wt* | A33/G | *wt* | 2 | ⬛⬛⬛⬛⬛⬛⬛⬛⬛⬛⬛⬛⬛⬛⬛⬛⬛⬛⬛⬛⬜⬜⬜⬜⬛⬛⬛⬛⬛⬛⬛⬛⬜⬜⬜⬜⬛⬛⬛⬛⬛⬛⬛ |
| 25 | F/30 | casa | Relapse | R | R | S | R | S | S | TCG531TTG | AGC315ACC | *wt* | *wt* | *wt* | A33/G | *wt* | 3 | ⬛⬛⬛⬛⬛⬛⬛⬛⬛⬛⬛⬛⬛⬛⬛⬛⬛⬛⬛⬛⬛⬛⬛⬛⬛⬛⬛⬛⬛⬛⬛⬛⬜⬜⬜⬜⬛⬛⬛⬛⬛⬛⬛ |
| 26 | M/NA | casa | Treatment after failure | R | R | S | S | R | S | TCG531TTG | AGC315ACC | ACC95AGC | *wt* | *wt* | A33/G | *wt* | 3 | ⬛⬛⬛⬛⬛⬛⬛⬛⬛⬛⬛⬛⬛⬛⬛⬛⬛⬛⬛⬛⬛⬛⬛⬛⬛⬛⬛⬛⬛⬛⬛⬛⬜⬜⬜⬜⬛⬛⬛⬛⬛⬛⬛ |
| 27 | F/40 | casa | Relapse | R | R | S | S | S | S | TCG531TTG | AGC315ACC | *wt* | *wt* | *wt* | A33/G | *wt* | 2 | ⬛⬛⬛⬛⬛⬛⬛⬛⬛⬛⬛⬛⬛⬛⬛⬛⬛⬛⬛⬛⬜⬜⬜⬜⬛⬛⬛⬛⬛⬛⬛⬛⬜⬜⬜⬜⬛⬛⬛⬛⬛⬛⬛ |
| 28 | M/NA | casa | Relapse | R | R | S | S | S | S | TCG531TTG | AGC315ACC | ACC95AGC | *wt* | *wt* | A33/G | *wt* | 2 | ⬛⬛⬛⬛⬛⬛⬜⬜⬛⬛⬛⬛⬛⬛⬛⬛⬛⬛⬛⬛⬜⬜⬜⬜⬛⬛⬛⬛⬛⬛⬛⬛⬜⬜⬜⬜⬛⬛⬛⬛⬛⬛⬛ |
| 29 | M/32 | casa | Relapse | R | R | S | S | S | S | TCG531TTG | AGC315ACC | *wt* | *wt* | *wt* | A33/G | *wt* | 2 | ⬛⬛⬛⬛⬛⬛⬛⬛⬛⬛⬛⬛⬛⬛⬛⬛⬛⬛⬛⬛⬛⬛⬛⬛⬛⬜⬜⬜⬜⬜⬜⬛⬜⬜⬜⬜⬛⬛⬛⬛⬛⬛⬛ |
| 30 | M/25 | casa | Relapse | S | R | S | S | S | R | TCG531TTG | AGC315ACC | *wt* | *wt* | *wt* | A33/G | *wt* | 3 | ⬛⬛⬛⬛⬛⬛⬜⬜⬛⬛⬛⬛⬛⬛⬛⬛⬛⬛⬛⬛⬜⬜⬜⬜⬛⬛⬛⬛⬛⬛⬛⬛⬜⬜⬜⬜⬛⬛⬛⬛⬛⬛⬛ |
| 31 | M/34 | casa | Treatmentafterfailure | R | R | S |  |  |  | TCG531TTG | AGC315ACC | *wt* | *wt* | *wt* | A33/G | *wt* | 2 | ⬛⬛⬛⬛⬛⬛⬛⬛⬛⬛⬛⬛⬛⬛⬛⬛⬛⬛⬛⬛⬜⬜⬜⬜⬛⬛⬛⬛⬛⬛⬛⬛⬜⬜⬜⬜⬛⬛⬛⬛⬛⬛⬛ |
| 32 | M/NA | casa | Relapse | R | R | S |  |  |  | TCG531TGG | AGC315ACC | ACC95AGC | *wt* | *wt* | A33/G | *wt* | 2 | ⬛⬛⬛⬛⬛⬛⬛⬛⬛⬛⬛⬛⬛⬛⬛⬛⬛⬛⬛⬛⬜⬜⬜⬜⬛⬛⬛⬛⬛⬛⬛⬛⬜⬜⬜⬜⬛⬛⬛⬛⬛⬛⬛ |
| 33 | M/NA | casa | Treatment after failure | R | R | S |  |  |  | CAC526ACC | AGC315ACC | *wt* | *wt* | *wt* | A33/G | *C14T* | 3 | ⬛⬛⬛⬛⬛⬛⬛⬛⬜⬜⬛⬛⬜⬛⬛⬛⬛⬛⬜⬛⬛⬛⬛⬛⬛⬛⬛⬛⬛⬛⬛⬛⬜⬜⬜⬜⬛⬛⬛⬛⬛⬛⬛ |
| 34 | M/36 | casa | Relapse | R | R | S |  |  |  | TCG531TTG | AGC315ACC | *wt* | *wt* | *wt* | A33/G | *wt* | 2 | ⬛⬛⬛⬛⬛⬛⬛⬛⬛⬛⬛⬛⬛⬛⬛⬛⬛⬛⬛⬛⬜⬜⬜⬜⬛⬛⬛⬛⬛⬛⬛⬛⬜⬜⬜⬜⬛⬛⬛⬛⬛⬛⬛ |
| 35 | M/40 | casa | Treatmentafterfailure | R | R | S |  |  |  | TCG531TTG | AGC315ACC | ACC95AGC | *wt* | *wt* | A33/G | *wt* | 2 | ⬛⬛⬛⬛⬛⬛⬛⬛⬛⬛⬛⬛⬛⬛⬛⬛⬛⬛⬛⬛⬜⬜⬜⬜⬛⬛⬛⬛⬛⬛⬛⬛⬜⬜⬜⬜⬛⬛⬛⬛⬛⬛⬛ |
| 36 | M/NA | casa | Treatment after failure | R | R | S | S | S | S | TCG531TTG | AGC315ACC | *wt* | *wt* | *wt* | A33/G | *C12T* | 3 | ⬛⬛⬛⬛⬛⬛⬛⬛⬜⬜⬛⬛⬜⬛⬛⬛⬛⬛⬜⬛⬛⬛⬛⬛⬛⬛⬛⬛⬛⬛⬛⬛⬜⬜⬜⬜⬛⬛⬛⬛⬛⬛⬛ |
| 37 | M/37 | casa | Relapse | R | R | S |  |  |  | TCG531TTG | AGC315ACC | *wt* | *wt* | *wt* | A33/G | *wt* | 2 | ⬛⬛⬛⬛⬛⬛⬛⬛⬛⬛⬛⬛⬛⬛⬛⬛⬛⬛⬛⬛⬛⬛⬛⬛⬛⬜⬜⬜⬜⬜⬜⬛⬜⬜⬜⬜⬛⬛⬛⬛⬛⬛⬛ |
| 38 | M/NA | casa | Treatmentafter failure | R | R | R | S | S | S | TCG531TTG | AGC315ACC | wt | *wt* | *wt* | wt | *wt* | 3 | ⬛⬛⬛⬛⬛⬛⬛⬛⬛⬛⬛⬛⬛⬛⬛⬛⬛⬛⬛⬛⬜⬜⬜⬜⬛⬛⬛⬛⬛⬛⬛⬛⬜⬜⬜⬜⬛⬛⬛⬛⬛⬛⬛ |
| 39 | M/NA | casa | Relapse | R | R | S |  |  |  | TCG531TTG | AGC315ACC | *wt* | *wt* | *wt* | A33/G | *wt* | 2 | ⬛⬛⬛⬛⬛⬛⬛⬛⬛⬛⬛⬛⬛⬛⬛⬛⬛⬛⬛⬛⬜⬜⬜⬜⬛⬛⬛⬛⬛⬛⬛⬛⬜⬜⬜⬜⬛⬛⬛⬛⬛⬛⬛ |
| 40 | M/NA | casa | Relapse | R | R | S |  |  |  | TCG531TTG | AGC315ACC | *wt* | *wt* | *wt* | A33/G | *wt* | 2 | ⬛⬛⬛⬛⬛⬛⬛⬛⬛⬛⬛⬛⬛⬛⬛⬛⬛⬛⬛⬛⬜⬜⬜⬜⬛⬛⬛⬛⬛⬛⬛⬛⬜⬜⬜⬜⬛⬛⬛⬛⬛⬛⬛ |
| 41 | M/29 | casa | Treatment after failure | S | R | S | S | S | S | TCG531TTG | AGC315ACC | *wt* | *wt* | *wt* | A33/G | *wt* | 2 | ⬛⬛⬛⬛⬛⬛⬛⬛⬛⬛⬛⬛⬛⬛⬛⬛⬛⬛⬛⬛⬜⬜⬜⬜⬛⬛⬛⬛⬛⬛⬛⬛⬜⬜⬜⬜⬛⬛⬛⬛⬛⬛⬛ |
| 42 | M/49 | casa | Treatment after failure | R | R | S | S | S | S | TCG531TTG | AGC315ACC | ACC95AGC | *wt* | *wt* | A33/G | *wt* | 2 | ⬛⬛⬛⬛⬛⬛⬛⬛⬛⬛⬛⬛⬜⬛⬛⬛⬛⬛⬛⬛⬜⬜⬜⬜⬛⬛⬛⬛⬛⬛⬛⬛⬜⬜⬜⬜⬛⬛⬛⬛⬛⬛⬛ |
| 43 | M/28 | casa | Treatment after failure | R | R | S |  | S | S | TCG531TTG | AGC315ACC | ACC95AGC | *wt* | *wt* | A33/G | *wt* | 2 | ⬛⬛⬛⬛⬛⬛⬛⬛⬛⬛⬛⬛⬛⬛⬛⬛⬛⬛⬛⬛⬛⬜⬜⬜⬛⬛⬛⬛⬛⬛⬛⬛⬜⬜⬜⬜⬛⬛⬛⬛⬛⬛⬛ |
| 44 | M/NA | casa | Treatment after failure | S | S | S | S | S | S | TCG531TTG | AGC315ACC | *wt* | *wt* | *wt* | A33/G | *wt* | 2 | ⬛⬛⬛⬛⬛⬛⬛⬛⬛⬛⬛⬛⬜⬛⬛⬛⬛⬛⬛⬛⬛⬛⬛⬛⬛⬛⬛⬛⬛⬛⬛⬛⬜⬜⬜⬜⬛⬛⬛⬜⬛⬛⬛ |
| 45 | M/42 | casa | Treatment after failure | R | R | S | S | S | S | TCG531TTG | AGC315ACC | ACC95AGC | *wt* | *wt* | A33/G | *wt* | 2 | ⬛⬛⬛⬛⬛⬛⬛⬛⬛⬛⬛⬛⬛⬛⬜⬜⬜⬛⬛⬛⬜⬜⬜⬜⬛⬛⬛⬛⬛⬛⬛⬛⬜⬜⬜⬜⬛⬛⬛⬛⬛⬛⬛ |
| 46 | M/NA | casa | Relapse | S | S | S | S | S | S | TCG531TTG | AGC315ACC | ACC95AGC | *wt* | *wt* | A33/G | *wt* | 2 | ⬛⬛⬛⬛⬛⬛⬛⬛⬛⬛⬛⬛⬛⬛⬛⬛⬛⬛⬛⬛⬜⬜⬜⬜⬛⬛⬛⬛⬛⬛⬛⬛⬜⬜⬜⬜⬛⬛⬛⬛⬛⬛⬛ |
| 47 | M/NA | casa | Relapse | R | S | S | S | S | S | TCG531TTG | AGC315ACC | *wt* | *wt* | *wt* | A33/G | *wt* | 2 | ⬛⬛⬛⬛⬛⬛⬛⬛⬛⬛⬛⬛⬛⬛⬛⬛⬛⬛⬛⬛⬛⬛⬛⬛⬛⬜⬜⬜⬜⬜⬜⬛⬜⬜⬜⬜⬛⬛⬛⬛⬛⬛⬛ |
| 48 | NA | casa |  | R | R | R | S | S | S | TCG531TTG | AGC315ACC | *wt* | *wt* | *wt* | A33/G | *wt* | 3 | ⬛⬛⬛⬛⬛⬛⬜⬜⬛⬛⬛⬛⬛⬛⬛⬛⬛⬛⬛⬛⬛⬛⬛⬛⬛⬛⬛⬛⬛⬛⬛⬛⬜⬜⬜⬜⬛⬛⬛⬛⬛⬛⬛ |
| 49 | NA | casa |  |  |  | R | S | S | S | TCG531TTG | AGC315ACC | *wt* | *wt* | *wt* | A33/G | *wt* | 3 | ⬛⬛⬛⬛⬛⬛⬛⬛⬛⬛⬛⬛⬜⬛⬛⬛⬛⬛⬛⬛⬜⬜⬜⬜⬛⬛⬛⬛⬛⬛⬛⬛⬜⬜⬜⬜⬛⬛⬛⬛⬛⬛⬛ |
| 50 | NA | casa |  |  |  | S | S | S | S | TCG531TTG | AGC315ACC | GAC94GCC | *wt* | *wt* | A33/G | *wt* | 3 | ⬛⬛⬛⬛⬛⬛⬛⬛⬛⬛⬛⬛⬛⬛⬛⬛⬛⬛⬛⬛⬜⬜⬜⬜⬛⬛⬛⬛⬛⬛⬛⬛⬜⬜⬜⬜⬛⬛⬛⬛⬛⬛⬛ |
| 51 | NA | casa |  |  |  | S | S | S | S | TCG531TTG | AGC315ACC | GAC94GCC; ACC95AGC | *wt* | *wt* | A33/G | *wt* | 3 | ⬛⬛⬛⬜⬜⬜⬜⬜⬜⬜⬜⬛⬜⬛⬛⬛⬛⬛⬛⬛⬛⬛⬛⬛⬛⬜⬜⬜⬜⬜⬜⬜⬜⬜⬜⬜⬜⬜⬜⬜⬜⬜⬜ |
| 52 | NA | casa |  |  |  | S | S | S | S | TCG531TTG | AGC315ACC | ACC95AGC | *wt* | *wt* | A33/G | *wt* | 2 | ⬜⬜⬜⬜⬜⬜⬜⬜⬜⬜⬜⬜⬜⬜⬜⬜⬜⬜⬜⬜⬜⬜⬜⬜⬜⬜⬜⬜⬜⬜⬜⬜⬜⬜⬛⬛⬛⬛⬛⬛⬛⬛⬛ |
| 53 | F/30 | casa |  |  |  |  |  |  |  | TCG531TTG | AGC315ACC | *wt* | *wt* | *wt* | A33/G | *wt* | 2 | ⬛⬛⬛⬛⬛⬛⬛⬛⬛⬛⬛⬛⬛⬛⬛⬛⬛⬛⬛⬛⬜⬜⬜⬜⬛⬛⬛⬛⬛⬛⬛⬛⬜⬜⬜⬜⬛⬛⬛⬛⬛⬛⬛ |
| 54 | F/24 | casa | Treatment after failure |  |  |  |  |  |  | TCG531TTG | AGC315ACC | *wt* | *wt* | *wt* | A33/G | *wt* | 2 | ⬛⬛⬛⬛⬛⬛⬛⬛⬛⬛⬛⬛⬛⬛⬛⬛⬛⬛⬛⬛⬜⬜⬜⬜⬛⬛⬛⬛⬛⬛⬛⬛⬜⬜⬜⬜⬛⬛⬛⬛⬛⬛⬛ |
| 55 | M/38 | casa | Relapse | R | R | S | S | S | R | TCG531TTG | AGC315ACC | *wt* | *wt* | *wt* | A33/G | *wt* | 3 | ⬛⬛⬛⬛⬛⬛⬛⬛⬛⬛⬛⬛⬛⬛⬛⬛⬛⬛⬛⬛⬛⬛⬛⬛⬛⬜⬜⬜⬜⬜⬜⬛⬜⬜⬜⬜⬛⬛⬛⬛⬛⬛⬛ |
| 56 | M/46 | casa | Relapse | R | R | S |  |  |  | TCG531TTG | AGC315ACC | *wt* | *wt* | *wt* | A33/G | *wt* | 2 | ⬛⬛⬛⬛⬛⬛⬛⬛⬛⬛⬛⬛⬛⬛⬛⬛⬛⬛⬛⬛⬜⬜⬜⬜⬛⬛⬛⬛⬛⬛⬛⬛⬜⬜⬜⬜⬛⬛⬛⬛⬛⬛⬛ |
| 57 | F/28 | casa |  | R | R | S |  |  |  | TCG531TTG | AGC315ACC | *wt* | *wt* | *wt* | A33/G | *wt* | 2 | ⬛⬛⬛⬛⬛⬛⬜⬜⬛⬛⬛⬛⬛⬛⬛⬛⬛⬛⬛⬛⬜⬜⬜⬜⬛⬛⬛⬛⬛⬛⬛⬛⬜⬜⬜⬜⬛⬛⬛⬛⬛⬛⬛ |
| 58 | M/30 | casa | Treatment after failure | R | S | S |  |  |  | TCG531TTG | AGC315ACC | *wt* | *wt* | *wt* | A33/G | *wt* | 2 | ⬛⬛⬛⬛⬛⬛⬛⬛⬛⬛⬛⬛⬛⬛⬛⬛⬛⬛⬛⬛⬜⬜⬜⬜⬛⬛⬛⬛⬛⬛⬛⬛⬜⬜⬜⬜⬛⬛⬛⬛⬛⬛⬛ |
| 59 | F/NA | casa | Treatment after failure | R | R | S |  |  |  | TCG531TTG | AGC315ACC | GAC94GCC | *wt* | *wt* | A33/G | *wt* | 3 | ⬛⬛⬛⬛⬛⬛⬛⬛⬛⬛⬛⬛⬛⬛⬛⬛⬛⬛⬛⬛⬜⬜⬜⬜⬛⬛⬛⬛⬛⬛⬛⬛⬜⬜⬜⬜⬛⬛⬛⬛⬛⬛⬛ |
| 60 | M/NA | casa | Relapse | R | R | S |  |  |  | TCG531TTG | AGC315ACC | *wt* | *wt* | *wt* | A33/G | *wt* | 2 | ⬛⬛⬛⬛⬛⬛⬜⬜⬛⬛⬛⬛⬛⬛⬛⬛⬛⬛⬛⬛⬜⬜⬜⬜⬛⬛⬛⬛⬛⬛⬛⬛⬜⬜⬜⬜⬛⬛⬛⬛⬛⬛⬛ |
| 61 | M/32 | casa | Treatment after failure | R | R | S |  |  |  | TCG531TTG | AGC315ACC | GAC94AAC | *wt* | *wt* | A33/G | *wt* | 3 | ⬛⬛⬛⬛⬛⬛⬛⬛⬛⬛⬛⬛⬜⬜⬜⬜⬛⬛⬛⬛⬛⬛⬛⬛⬛⬛⬛⬛⬛⬛⬛⬛⬜⬜⬜⬜⬛⬛⬛⬛⬛⬛⬛ |
| 62 | F/23 | casa |  | R | R | S |  |  |  | TCG531TTG | AGC315ACC | *wt* | *wt* | *wt* | A33/G | *wt* | 2 | ⬛⬛⬛⬛⬛⬛⬛⬛⬛⬛⬛⬛⬛⬛⬛⬛⬛⬛⬛⬛⬜⬜⬜⬜⬛⬛⬛⬛⬛⬛⬛⬛⬜⬜⬜⬜⬛⬛⬛⬛⬛⬛⬛ |
| 63 | M/29 | casa | Treatment after failure | R | S | S |  |  |  | TCG531TTG | AGC315ACC | *wt* | *wt* | *wt* | A33/G | *wt* | 2 | ⬛⬛⬛⬛⬛⬛⬛⬛⬛⬛⬛⬛⬛⬛⬛⬛⬛⬛⬛⬛⬜⬜⬜⬜⬛⬛⬛⬛⬛⬛⬛⬛⬜⬜⬜⬜⬛⬛⬛⬛⬛⬛⬛ |
| 64 | F/NA | casa |  | R | R | S |  |  |  | TCG531TTG | AGC315ACC | *wt* | *wt* | *wt* | A33/G | *wt* | 2 | ⬛⬛⬛⬛⬛⬛⬜⬜⬛⬛⬛⬛⬛⬛⬛⬛⬛⬛⬛⬛⬜⬜⬜⬜⬛⬛⬛⬛⬛⬛⬛⬛⬜⬜⬜⬜⬛⬛⬛⬛⬛⬛⬛ |
| 65 | M/NA | casa | New patient | R | R | S |  |  |  | TCG531TTG | AGC315ACC | GAC94GCC | *wt* | *wt* | A33/G | *wt* | 3 | ⬛⬛⬛⬛⬛⬛⬛⬛⬛⬛⬛⬛⬛⬛⬛⬛⬛⬛⬛⬛⬜⬜⬜⬜⬛⬛⬛⬛⬛⬛⬛⬛⬜⬜⬜⬜⬛⬛⬛⬛⬛⬛⬛ |
| 66 | F/NA | casa |  | S | S | S |  |  |  | GAC516GTC | AGC315ACC | *wt* | *wt* | *wt* | A33/G | *wt* | 2 | ⬛⬛⬛⬛⬛⬛⬛⬛⬛⬛⬛⬛⬛⬛⬛⬛⬛⬛⬛⬛⬜⬜⬜⬜⬛⬛⬛⬛⬛⬛⬛⬛⬜⬜⬜⬜⬛⬛⬛⬛⬛⬛⬛ |
| 67 | M/42 | casa | New patient |  |  |  |  |  |  | TCG531TTG | AGC315ACC | GAC94GCC | *wt* | *wt* | A33/G | *wt* | 3 | ⬜⬜⬜⬜⬜⬜⬜⬜⬜⬜⬜⬜⬜⬜⬜⬜⬜⬜⬜⬜⬜⬜⬜⬜⬜⬜⬜⬜⬜⬜⬜⬜⬜⬜⬛⬛⬛⬛⬛⬛⬛⬛⬛ |
| 68 | M/34 | casa | Treatment after failure | S | S | S |  |  |  | GAC516GTC | AGC315ACC | *wt* | *wt* | *wt* | A33/G | *wt* | 2 | ⬛⬛⬛⬛⬛⬛⬛⬛⬛⬛⬛⬛⬛⬛⬛⬛⬛⬛⬛⬛⬛⬛⬛⬛⬛⬛⬛⬛⬛⬛⬛⬛⬜⬜⬜⬜⬛⬛⬛⬛⬛⬛⬛ |
| 69 | M/42 | casa | Treatment after failure | R | R | S |  |  |  | TCG531TTG | AGC315ACC | ACC95AGC | *wt* | *wt* | A33/G | *wt* | 2 | ⬛⬛⬛⬛⬛⬛⬜⬜⬛⬛⬛⬛⬛⬛⬛⬛⬛⬛⬛⬛⬛⬛⬛⬛⬛⬛⬛⬛⬛⬛⬛⬛⬜⬜⬜⬜⬛⬛⬛⬛⬛⬛⬛ |
| 70 | M/34 | casa | Relapse | R | R | S |  |  |  | TCG531TTG | AGC315ACC | GCG90GTG | *wt* | *wt* | A33/G | *wt* | 3 | ⬛⬛⬛⬛⬛⬛⬜⬜⬛⬛⬛⬛⬛⬛⬛⬛⬛⬛⬛⬛⬜⬜⬜⬜⬛⬛⬛⬛⬛⬛⬛⬛⬜⬜⬜⬜⬛⬛⬛⬛⬛⬛⬛ |
